# *QuickStats:* Percentage* of Adults Aged ≥18 Years Who Reported Having a Severe Headache or Migraine in the Past 3 Months,^†^ by Sex and Age Group — National Health Interview Survey,^§^ United States, 2015

**DOI:** 10.15585/mmwr.mm6624a8

**Published:** 2017-06-23

**Figure:**
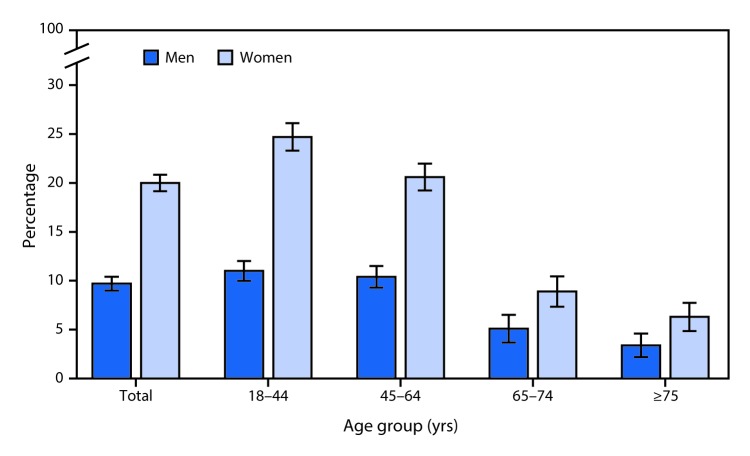
In 2015, 20.0% of women and 9.7% of men aged ≥18 years had a severe headache or migraine in the past 3 months. Overall and for each age group, women aged ≥18 years were more likely than men to have had a severe headache or migraine in the past 3 months. For both sexes, a report of a severe headache or migraine in the the past 3 months decreased with advancing age, from 11.0% among men aged 18–44 years to 3.4% among men aged ≥75 years and from 24.7% among women aged 18–44 years to 6.3% among women aged ≥75 years.

**Source:** National Health Interview Survey, 2015. https://www.cdc.gov/nchs/nhis/index.htm.

